# Piperine as potential therapy of post-weaning porcine diarrheas: an in vitro study using a porcine duodenal enteroid model

**DOI:** 10.1186/s12917-022-03536-6

**Published:** 2023-01-09

**Authors:** Saravut Satitsri, Nattaphong Akrimajirachoote, Kanokkan Nunta, Nitwarat Ruennarong, Orawan Amnucksoradej, Chatchai Muanprasat

**Affiliations:** 1 Chakri Naruebodindra Medical Institute, Faculty of Medicine Ramathibodi Hospital, Mahidol University, Bang Phli, Samut Prakarn, 10540 Thailand; 2 Department of Physiology, Faculty of Veterinary Medicine, Kasetsart University, Bangkok, 10900 Thailand; 3 Vet Products Research and Innovation Center Co., Ltd., Pathum Thani, 12120 Thailand

**Keywords:** Weaning piglet, Diarrhea, Intestinal inflammation, Oxidative stress, Porcine enteroid

## Abstract

Post-weaning diarrhea in piglets is a major problem, resulting in a significant loss in pig production. This study aimed to investigate the effects of piperine, an alkaloid abundantly found in black peppers, on biological activities related to the pathogenesis of post-weaning diarrhea using a porcine duodenal enteroid model, a newly established intestinal stem cell-derived in vitro model recapitulating physiology of porcine small intestinal epithelia. Porcine duodenal enteroid models were treated with disease-relevant pathological inducers with or without piperine (8 μg/mL and/or 20 μg/mL) before measurements of oxidative stress, mRNA, and protein expression of proinflammatory cytokines, nuclear factor-kappa B (NF-κB) nuclear translocation, barrier leakage, and fluid secretion. We found that piperine (20 μg/mL) inhibited H_2_O_2_-induced oxidative stress, TNF-α-induced mRNA, and protein expression of proinflammatory cytokines without affecting NF-κB nuclear translocation, and prevented TNF-α-induced barrier leakage in porcine duodenal enteroid monolayers. Importantly, piperine inhibited fluid secretion induced by both forskolin and heat-stable toxins (STa) in a three-dimensional model of porcine duodenal enteroids. Collectively, piperine possesses both anti-inflammatory and anti-secretory effects in porcine enteroid models. Further research and development of piperine may provide novel interventions for the treatment of post-weaning porcine diarrhea.

**Supplementary Information:**

The online version contains supplementary material available at 10.1186/s12917-022-03536-6.

## Introduction

Post-weaning diarrhea (PWD) is a major problem in pig production, causing economic loss, lower growth performance, and an increased mortality rate [1, 2]. The mortality rate caused by PWD may reach approximately 20-30% [1]. The pathophysiology of PWD involves excessive intestinal fluid secretion, resulting in severe fluid loss [3]. This pathological process is driven by transepithelial chloride secretion via cystic fibrosis transmembrane conductance regulators (CFTR), a cAMP-regulated chloride channel [4, 5]. Moreover, weaning piglets are vulnerable to *Escherichia coli* infection, causing more severe symptoms by promoting CFTR-mediated chloride secretion via effects of bacterial enterotoxins, e.g., heat-stable toxins (STa) and intestinal barrier disruption via oxidative stress and proinflammatory responses [6]. Notably, tumor necrosis factor-α (TNF-α), a proinflammatory cytokine elevated in the intestine-weaning piglet, plays important role in eliciting inflammatory responses and associated pathogenesis in PWD [7]. At present, antibiotics prescribed for medical conditions in humans, including colistin, have been used to treat PWD [8], with significant concern for promoting the emergence of antibiotic-resistant bacteria. Therefore, novel therapeutic measures for PWD are in urgent need, particularly those targeting pathological processes in hosts, including oxidative stress, inflammation-associated barrier dysfunction, and intestinal fluid secretion.

Black peppers (*Piper nigrum L.*) are used as spice and seasoning in the household. Piperine, an alkaloid serving as the chief chemical compound in black peppers, has been demonstrated to possess several biological activities, including anti-oxidative stress and anti-inflammation [9–11]. Interestingly, we have recently reported that piperine has anti-secretory effects in human intestinal epithelial (T84) cell lines by inhibiting CFTR, calcium-activated chloride channels (CaCC), and calcium-activated potassium channels [12]. Interestingly, piperine supplement promotes growth performance as well as meat quality in pigs [13]. However, the effects of piperine on oxidative stress, inflammatory responses, and fluid secretion related to PWD pathogenesis in porcine intestinal epithelia are unexplored.

Enteroids or mini-guts are a powerful and physiologically relevant model of intestinal epithelia recently developed from intestinal stem cells residing in the intestinal crypts [14]. Enteroids contain all types of cells in intestinal epithelia, which exhibit functional characteristics of each intestinal region in vivo dependent on areas from which intestinal stem cells/crypts are isolated. Enteroids from mice, humans, and pigs have recently been established for investigating both intestinal functions and pathogenesis using either two-dimensional (2D; monolayers) or three-dimensional (3D) models [15–17]. Interestingly, a swelling (3D) assay has been developed and proven useful to evaluate anti-secretory effects [18, 19]. This study utilized 2D and 3D porcine enteroids generated from duodenal crypts isolated from weaned pigs to explore the effects of piperine on oxidative stress, inflammation-associated barrier breach, and fluid secretion associated with PWD pathogenesis.

## Materials and methods

### Ethics statement

This study has been approved by the Institutional Animal Care and Use Committee of the Faculty of Veterinary Medicine, Kasetsart University (permit number ACKU64-VET-064). Leftover samples from this study were collected in accordance with the recommendations in the Guide for the Care and Use of Laboratory Animals of the National Institutes of Health, U.S.A.

### Cell lines

L-WRN cells were from the American Type Culture Collection (ATCC) (catalog number CRL-3276; Manassas, VA, USA). The cells were maintained by Dulbecco’s Modified Eagle Medium (DMEM) high glucose supplemented with 0.5 mg/mL of G418 (catalog number 11811031, Gibco, Waltham, MA, USA) and 0.5 mg/mL of Hygromycin B (catalog number 10687010, Gibco, Waltham, MA, USA). The conditioned medium was prepared according to ATCC’s guidelines.

### Materials

CFTR_inh_-172 and 4,4′-diisothiocyanatostilbene-2,2 ´-disulfonic acid disodium salt hydrate (DIDS) were purchased from Merck (Darmstadt, Germany). GlyH-101 was obtained from R&D Systems (Minneapolis, MN, USA). Piperine (purity > 98%) was prepared from black peppers as previously described [20].

### Crypt isolation for porcine enteroid culture

Three weeks after weaning, piglets were acquired from the Faculty of Veterinary Medicine, Kasetsart University. Porcine duodenums as leftover samples were collected and crypt isolation was performed with some modifications as previously described [21]. Briefly, porcine duodenal tissues were cut into small pieces. Porcine duodenal tissues were then washed with a chelated solution containing 5.55 mM Na_2_HPO_4_, 7.9 mM KH_2_PO_4_, 95.82 mM NaCl, 1.6 mM KCl, 43.82 mM sucrose, 54.89 mM D-sorbitol, and 0.5 mM dithiothreitol. Crypts from porcine duodenal tissue were subsequently isolated at 0.5 M EDTA. Crypts were formed into 3D porcine duodenal enteroids in Matrigel (catalog number 356237, Corning, NY, USA) mixed with enteroid-growing media containing L-WRN-conditioned media, 1X B27, 1 mM N-acetyl cysteine, 100 μg/mL Primocin, 50 ng/mL EGF, 10 nM Gastrin, 10 μM SB202190, and 500 nM A83-01. Porcine duodenal enteroids after more than 10 passages were used for the experiments.

### Oxidative stress measurement

It is known that H_2_O_2_ induces oxidative stress via the Fenton reaction between H_2_O_2_ and Fe^2+^ generating hydroxyl radicals, a type of reactive oxygen species (ROS) [22]. Therefore, H_2_O_2_ was used as an inducer of oxidative stress in this study. Oxidative stress was measured using 2′,7′-dichlorofluorescin diacetate (DCFDA) assays (catalog number D6883, Sigma Aldrich, Burlington, MA, USA), which detected reactive oxygen species (ROS) inside the cells. The 2D porcine duodenal enteroids were seeded onto 24-well plates. Cells were incubated for 2 h in serum-free DMEM high glucose media containing 1 mM H_2_O_2_ with or without piperine (8 μg/mL or 20 μg/mL). Fluorescence intensity (485 nm/ 530 nm) was measured using the Synergy Neo2 Plate Reader (Biotek, Santa Clara, CA, USA.). Trolox at 2 mM (catalog number 238813, Sigma Aldrich, Burlington, MA, USA) was used as a positive control.

### Intestinal barrier integrity evaluation

Intestinal barrier integrity of 2D porcine duodenal enteroid monolayers was evaluated using fluorescein isothiocyanate (FITC)-dextran (4 kDa) flux assays. Cells were seeded onto 24-well inserts (catalog number 3470, Corning, NY, USA) with collagen type IV coating (catalog number C6725, Sigma Aldrich, Burlington, MA, USA). Cells were maintained for at least 14 days, when transepithelial electrical resistance was ~ 500 Ω.cm^2^. In this experiment, enteroid monolayers were treated for 24 h with TNFα (50 ng/mL) with or without piperine (8 μg/mL or 20 μg/mL) before the addition of 15 μL of 10 mg/mL FITC-dextran (4 kDa) (catalog number 46944, Sigma Aldrich, Burlington, MA, USA). An hour later, 100 μL of basolateral media was collected for measuring fluorescence intensity (495 nm/519 nm) using the Synergy Neo2 Plate Reader (Biotek, Santa Clara, CA, USA). Concentrations of FITC-dextran in the collected media were calculated using the standard curve of FITC-dextran with known concentrations.

### Immunofluorescence staining of NF-κB nuclear translocation and ZO-1 localization

2D porcine duodenal enteroids were seeded onto 24-well plates. Cells were then treated for 30 min with TNFα (50 ng/mL) [23] with or without piperine (8 μg/mL or 20 μg/mL), followed by 1-h fixation with 4% paraformaldehyde, washing with PBS, and 1-h blocking and permeabilization with 0.1% Triton X-100 and 1% bovine serum albumin in PBS. Fixed cells were incubated overnight with rabbit NF-κB p65 antibody (catalog number D14E12, cell signaling, Danvers, MA, USA), washed with PBS, incubated for an hour with Goat anti-Rabbit IgG (H + L) Highly Cross-Adsorbed Secondary Antibody, Alexa Fluor 488 (catalog number A-11034, Thermo Fisher Scientific, Waltham, MA, USA), and counterstained for 10 min with Hoechst (catalog number H3570, Invitrogen, Waltham, MA, USA) for nuclear staining. The images were captured by a fluorescence microscope (Nikon Eclipse Ts2R inverted fluorescence microscope, Tokyo, Japan). Localization of NF-κB p65 and nuclear staining was analyzed using Fiji ImageJ [24]. For ZO-1 localization, 2D porcine duodenal enteroids were seeded onto 24-well plates. Cells were then treated for 24 h with TNFα (50 ng/mL) with or without piperine (8 μg/mL or 20 μg/mL), followed by 1-h fixation with 4% paraformaldehyde, washing with PBS, and 1-h blocking and permeabilization with 0.1% Triton X-100 and 1% bovine serum albumin in PBS. Fixed cells were incubated overnight with rabbit ZO-1 antibody (catalog number ab96587, Abcam, Cambridge, United Kingdom), washed with PBS, incubated for an hour with Goat anti-Rabbit IgG (H + L) Highly Cross-Adsorbed Secondary Antibody, Alexa Fluor 488 (catalog number A-11034, Thermo Fisher Scientific, Waltham, MA, USA), and counterstained for 10 min with Hoechst (catalog number H3570, Invitrogen, Waltham, MA, USA) for nuclear staining. Images were captured using a confocal microscope (Opera Phenix Plus High-Content Screening System, Perkin Elmer, Waltham, MA, USA).

### Quantitative real-time PCR

2D porcine duodenal enteroids were seeded onto 24-well plates. Cells were treated for 24 h with TNFα (50 ng/mL) with or without piperine (8 μg/mL or 20 μg/mL) and collected for RNA isolation using Monarch Total RNA Miniprep Kit (NEB, Ipswich, MA, USA). The quantity of isolated RNA was measured by nanophotometer (Implen NP80, Munich, Germany). Isolated RNA was converted into cDNA using Reverse Transcription Kits (Biorad, Hercules, CA, USA). Quantitative real-time PCR was performed using CFX96 Touch Real-Time PCR (Biorad, Hercules, CA, USA) with iTaq universal SYBR green supermix (Biorad, Hercules, CA, USA) for DNA amplicon measurement under the following conditions: 95 °C 5 min; 40 cycles of 60 °C for 30 s. Primers of target genes (Table 1) were synthesized by Bio Basic (Singapore). Target genes were normalized to GAPDH. The calculation of mRNA expression was performed using the ddCT method.

**Table 1:** Primers of target genes analyzed by real-time PCR

Genes	Primer sequence (5′-3′)
TNF-α	FW: TGCCTACTGCACTTCGAGGTTATC
	RW: CAGATAAGCCCGTCGCCCAC
IL-1β	FW: AATTCGAGTCTGCCCTGTACCC
	RW: GCCAAGATATAACCGACTTCACCA
IL-6	FW: CAGAGATTTTGCCGAGGATG
	RW: TGGCTACTGCCTTCCCTACC
IL-8	FW: GACCCCAAGGAAAAGTGGGT
	RW: TGACCAGCACAGGAATGAGG
LGR5	FW: CCTTGGCCCTGAACAAAATA
	RW: ATTTCTTTCCCAGGGAGTGG
Lysozyme	FW: GGTCTATGATCGGTGCGAGT
	RW: AACTGCTTTGGGTGTCTTGC
SGLT1	FW: TCACCAAGCCCATTCCAGATG
	RW: GCTTCTTGAATGTCCTCCTCCT
GAPDH	FW: ATGGTGAAGGTCGGAGTGAA
	RW: CGTGGGTGGAATCATACTGG

### Immunoblotting

2D porcine duodenal enteroids were seeded onto 24-well plates. Cells were treated for 24 h with TNFα (50 ng/mL) with or without piperine (8 μg/mL or 20 μg/mL) and collected for protein using RIPA buffer (Thermo Fisher Scientific, Waltham, MA, USA) with protease inhibitor (catalog number 05892970001, Roche, Basel, Switzerland) and phosphatase inhibitor (Roche, Basel, Switzerland). Proteins were loaded into SDS-polyacrylamide gel and were transferred into a nitrocellulose membrane (catalog number 1620115, Biorad, Hercules, CA, USA). The membrane was divided into sections based on the molecular weight of the proteins before being occluded with 5% non-fat dry milk for an hour. Proteins were then incubated overnight with rabbit IL-1β polyclonal antibody (catalog number ab9722, Abcam, Cambridge, UK) or rabbit β-actin antibody for another membrane (catalog number 4970, cell signaling, Danvers, MA, USA). Tris-Boric Saline plus 0.05% Tween 20 was used for membrane washing. Membranes were incubated with anti-rabbit IgG conjugated to horseradish peroxidase (Abcam, Cambridge, UK), and submerged with Luminata Forte Western HRP substrate (Merck Millipore, Darmstadt, Germany). Membranes were exposed to ChemiDoc (Biorad, Hercules, CA, USA). The band intensity was analyzed using Fiji ImageJ [24].

### Swelling assay

Matrigel was used to seed 3D porcine duodenal enteroids onto 48-well plates. Cells were then treated with piperine (20 μg/mL), CFTR_inh_-172 (20 μM), GlyH-101 (50 μM), or DIDS (200 μM) for 1 h and were followed by an addition of forskolin (5 μM) or STa toxin (100 nM). Images were captured every 5 minutes in the area that contained at least 10 enteroids per area using Cytation 5 Cell Imaging Multi-Mode Reader (Biotek, Santa Clara, CA, USA). Time-lapse imaging was performed to monitor enteroid swelling at 5 frames per second. Areas of each enteroid in the images were measured using Fiji ImageJ [24].

### Data analysis

Data are expressed as means ± S.E.M. Each group was compared and analyzed using one-way analysis of variance (ANOVA) followed by Tukey’s post hoc test. The *p*-value of < 0.05 was considered statistically significant. All data were analyzed in GraphPad Prism 5 (La Jolla, CA, USA).

## Results

### Establishment and characterization of porcine duodenal enteroids

**Fig. 1:**
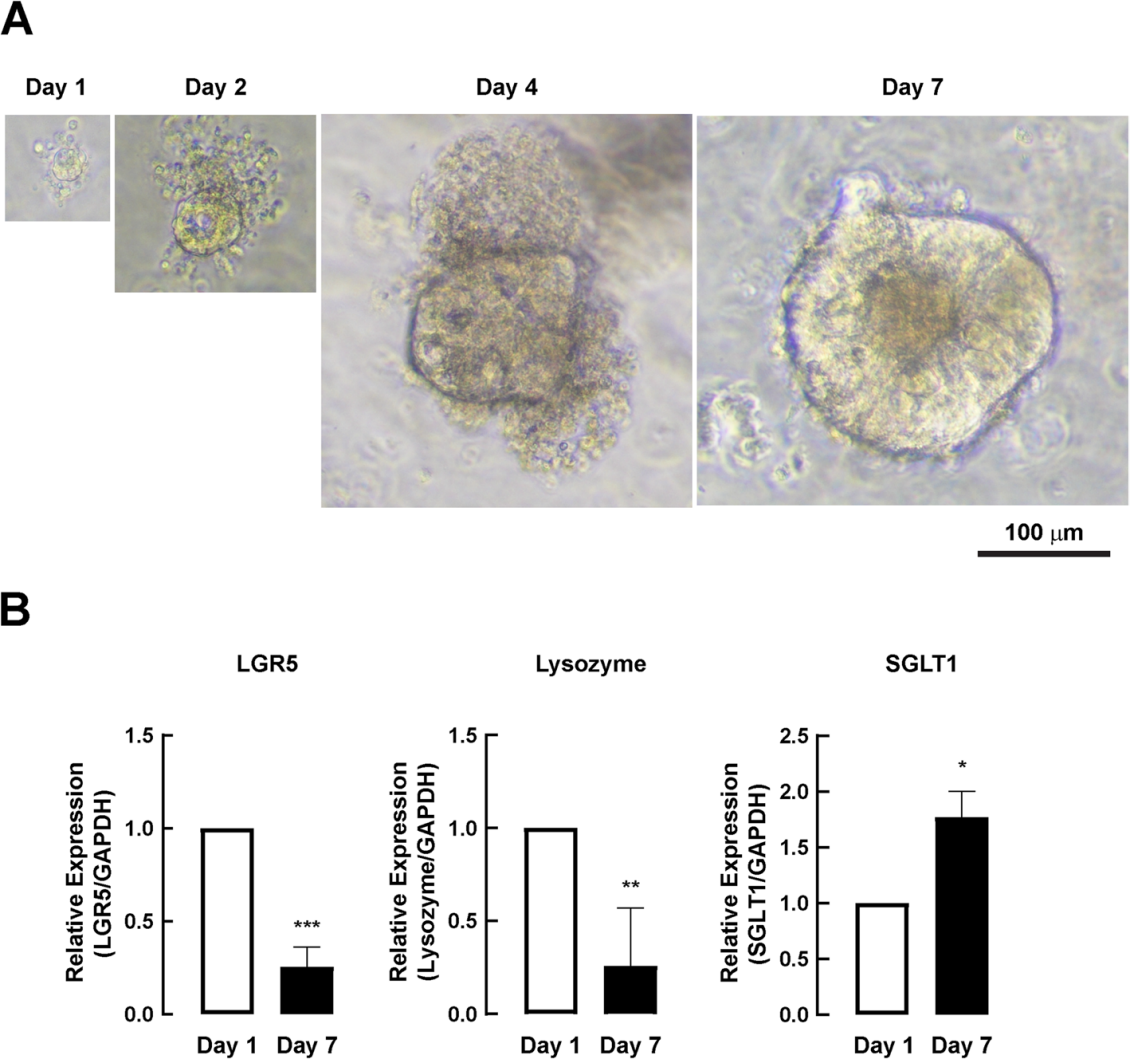
The establishment and characterization of porcine duodenal enteroid. **a** Representative images of porcine duodenal enteroid at day 1, day 2, day 4, and day 7 after enteroid splitting were depicted. Scale bar equaled to 100 μm. **b** mRNA expression of intestinal markers (LGR5, lysozyme, and SGLT1) in 3D porcine duodenal enteroid at day 1 and day 7 after splitting (*n* = 4) (*: *p*-value < 0.05 compared with day 1, **: *p*-value < 0.01 compared with day 1, ***: *p*-value < 0.001 compared with day 1)

The ability to produce enteroids using porcine small intestine crypts has been proven [15]. In this study, porcine duodenal enteroids from weaning piglets were successfully established. As shown in Fig. 1A, porcine duodenal enteroids exhibited gradual growth from day 1 to day 7 after enteroid splitting. Furthermore, mRNA expression of intestinal stem cell markers including leucine-rich repeat-containing G-protein-coupled receptor 5 (LGR5) (a marker of crypt base columnar cells) and lysozyme (a marker of Paneth cells) was detected on day 1, which was downregulated at day 7 (Fig. 1B). In contrast, mRNA expression of sodium-dependent glucose cotransporters 1 (SGLT1) (a marker of differentiated duodenal epithelial cells) on day 7 was significantly higher than that on day 1 (Fig. 1B). These results indicate the successful establishment of porcine duodenal enteroids in our study.

### Effect of piperine on H_2_O_2_-induced oxidative stress on 2D porcine duodenal enteroids

**Fig. 2:**
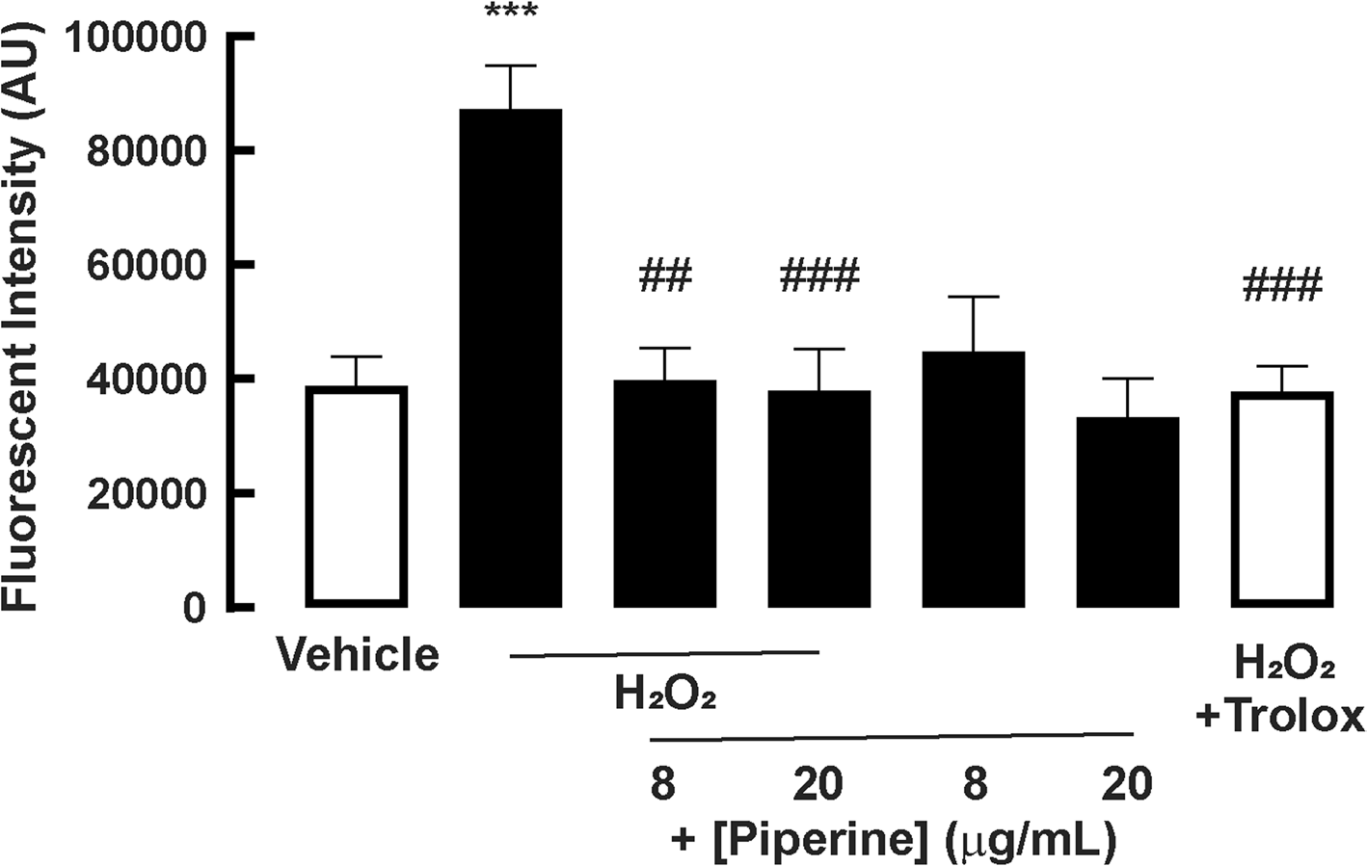
The effect of piperine on H_2_O_2_-induced oxidative stress in 2D porcine duodenal enteroid. 2′,7′-Dichlorofluorescin diacetate, (Reactive Oxygen Species (ROS)-sensitive dye, was incubated for 1 hour in 2D porcine duodenal enteroid. After incubation, cells were treated with vehicle or 1 mM H_2_O_2_ with or without 8 or 20 μg/mL piperine. 2 mM Trolox was also used as positive control (*n* = 5) (***: *p*-value < 0.001 compared with vehicle, ##: *p*-value < 0.01 compared with 1 mM H_2_O_2_, ###: *p*-value < 0.001 compared with 1 mM H_2_O_2_)

To investigate the anti-oxidative effects of piperine in 2D porcine duodenal enteroids, DCFDA-based ROS assays were performed after the H_2_O_2_ challenge with or without co-treatment with piperine. As demonstrated in Fig. 2, H_2_O_2_ treatment significantly increased DCFDA fluorescence intensity, indicating an increase in ROS. Interestingly, co-treatment with piperine at low (8 μg/mL) and high (20 μg/mL) concentrations of piperine completely abolished H_2_O_2_-induced ROS generation, with the use of Trolox as a positive control. Piperine alone at both concentrations had no effect on ROS. This result indicates that piperine exerts an anti-oxidative effect in 2D porcine duodenal enteroid.

### Effects of piperine on TNF-α-induced inflammatory responses in 2D porcine duodenal enteroid

**Fig. 3:**
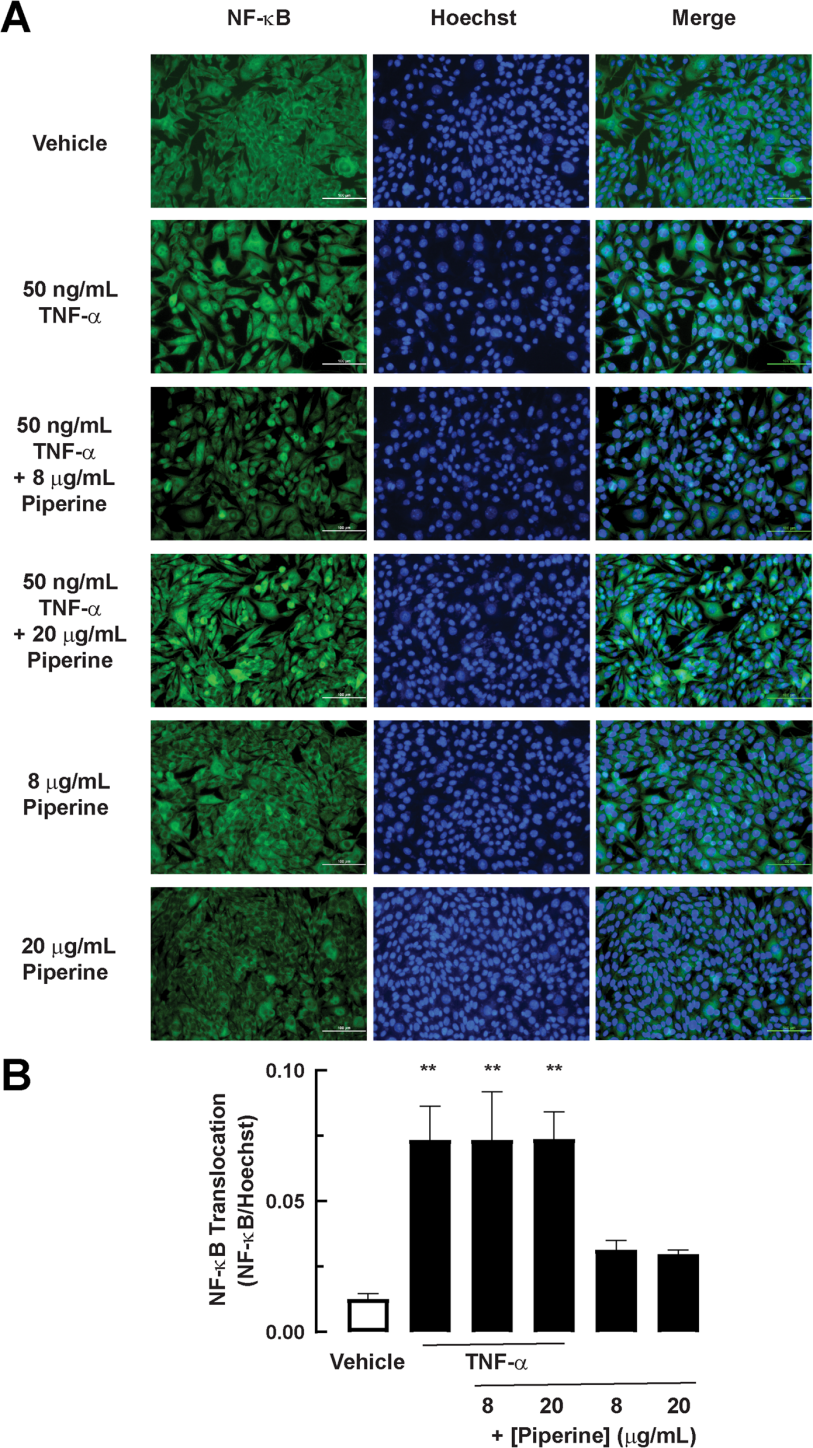
The effect of piperine on TNF-α-induced NF-κB translocation in 2D porcine duodenal enteroid. **a** The representative image of 2D porcine duodenal enteroid. Cells were treated with 50 ng/mL TNF-α with or without 8 or 20 μg/mL piperine for 30 minutes. Cells were stained with rabbit NF-κB antibody, following with Alexa Fluor 488 anti-rabbit IgG and Hoechst as nuclear staining. **b** Positive localization of NF-κB staining inside nuclear staining was counted divided by the amount of nuclear staining. One sample was measured at least 5 areas (*n* = 3) (**: *p*-value < 0.01 compared with vehicle)

Apart from oxidative stress, the elevation of TNF-α levels and inflammatory responses was found in the intestinal tissues of weaning piglets [7]. To investigate the effect of piperine on TNF-α-induced NF-κB activation, NF-κB nuclear translocation was analyzed using immunofluorescence staining after 30-min treatment with TNF-α (50 ng/mL) in the presence or absence of piperine in a 2D model of porcine duodenal enteroids. As shown in Fig. 3A and B, TNF-α induced NF-κB nuclear translocation, which was unaffected by co-treatment with piperine at 8 μg/mL and 20 μg/mL. This result indicates that piperine does not inhibit TNF-α-induced NF-κB activation.

**Fig. 4:**
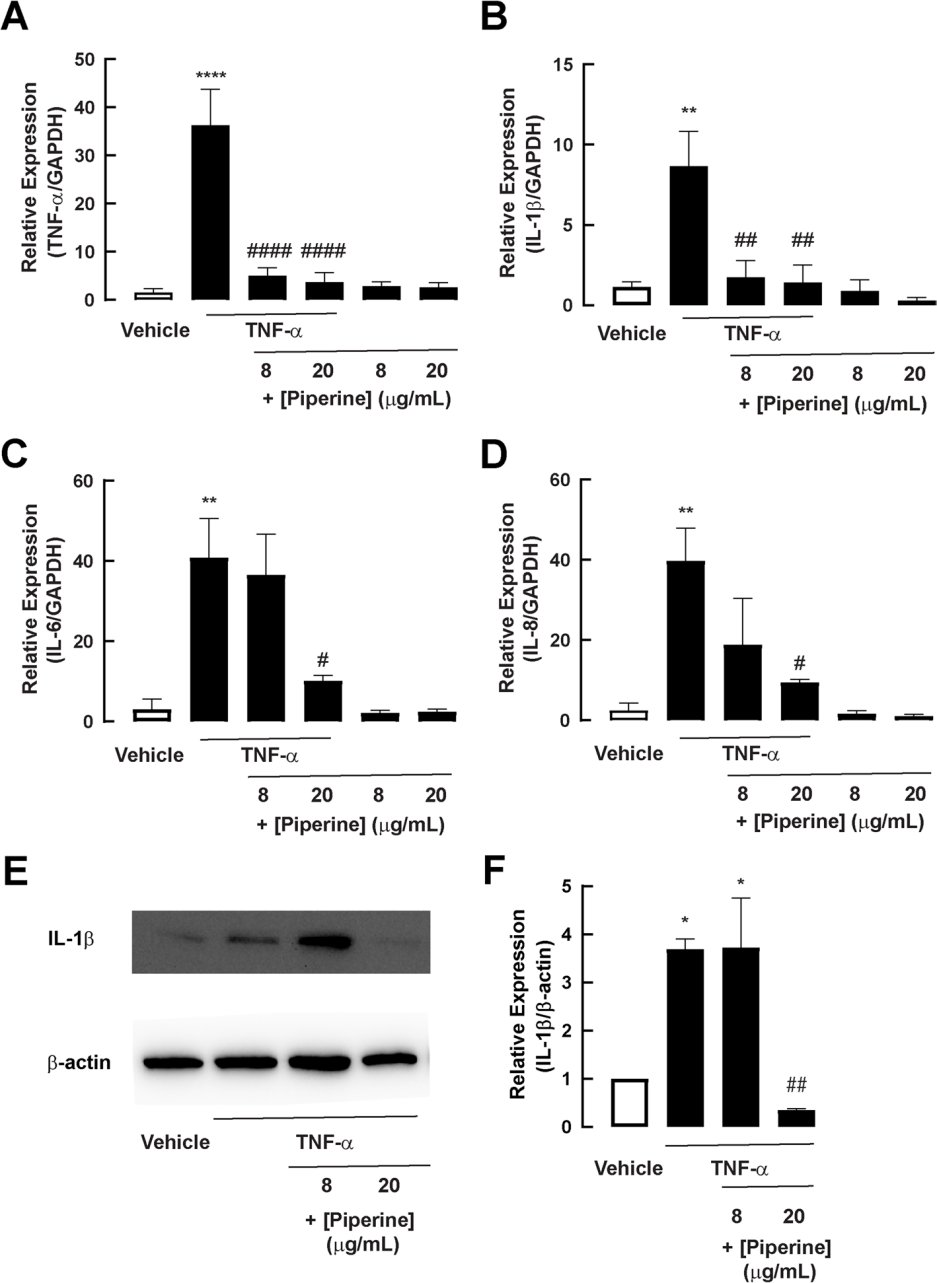
The effect of piperine on mRNA of TNF-α-induced proinflammatory cytokines in 2D porcine duodenal enteroid. Cells were treated with 50 ng/mL TNF-α with or without 8 or 20 μg/mL piperine for 24 hours. Cell were harvested for mRNA expression. GAPDH was measured as housekeeping gene. **a** The mRNA expression of TNF-α in 2D porcine duodenal enteroid (*n* = 4) (****: *p*-value < 0.0001 compared with vehicle, ####: *p*-value < 0.0001 compared with 50 ng/mL TNF-α. (**b**) The mRNA expression of IL-1β in 2D porcine duodenal enteroid (*n* = 4)(**: *p*-value < 0.01 compared with vehicle, ##: *p*-value < 0.01 compared with 50 ng/mL TNF-α). **c** The mRNA expression of IL-6 in 2D porcine duodenal enteroid (*n* = 4) (**: *p*-value < 0.01 compared with vehicle, #: *p*-value < 0.05 compared with 50 ng/mL TNF-α). **d** The mRNA expression of IL-8 in 2D porcine duodenal enteroid (*n* = 4) (**: *p*-value < 0.01 compared with vehicle, #: *p*-value < 0.05 compared with 50 ng/mL TNF-α). **e** The representative images of protein expression of IL-1β and β-actin in 2D porcine duodenal enteroid. **f** The protein expression of IL-1β in 2D porcine duodenal enteroid (*n* = 3) (*: *p*-value < 0.05 compared with vehicle, ##: *p*-value < 0.01 compared with 50 ng/mL TNF-α)

We next investigated the effect of piperine on TNF-α-induced inflammatory responses by measuring mRNA expression of proinflammatory cytokines (TNF-α, IL-1β, IL-6, and IL-8) after a 24-h challenge with TNF-α with or without co-treatment with piperine in a 2D model of porcine duodenal enteroids. As depicted in Fig. 4A and B, piperine at 8 μg/mL significantly suppressed mRNA expression of TNF-α and IL-1β. Interestingly, piperine at 20 μg/mL significantly inhibited the expression of all four proinflammatory cytokines (Fig. 4C and D). Furthermore, protein expression of IL-1β was determined to confirm the anti-inflammatory effect of piperine in porcine duodenal enteroids. As depicted in Fig. 4E and F, piperine at 20 μg/mL significantly inhibited the TNF-α-induced protein expression of IL-1β. These data indicate that piperine possesses an anti-inflammatory effect against TNF-α in 2D porcine duodenal enteroids*.*

### Effect of piperine on TNF-α-induced intestinal barrier leakage in 2D porcine duodenal enteroid monolayer

**Fig. 5:**
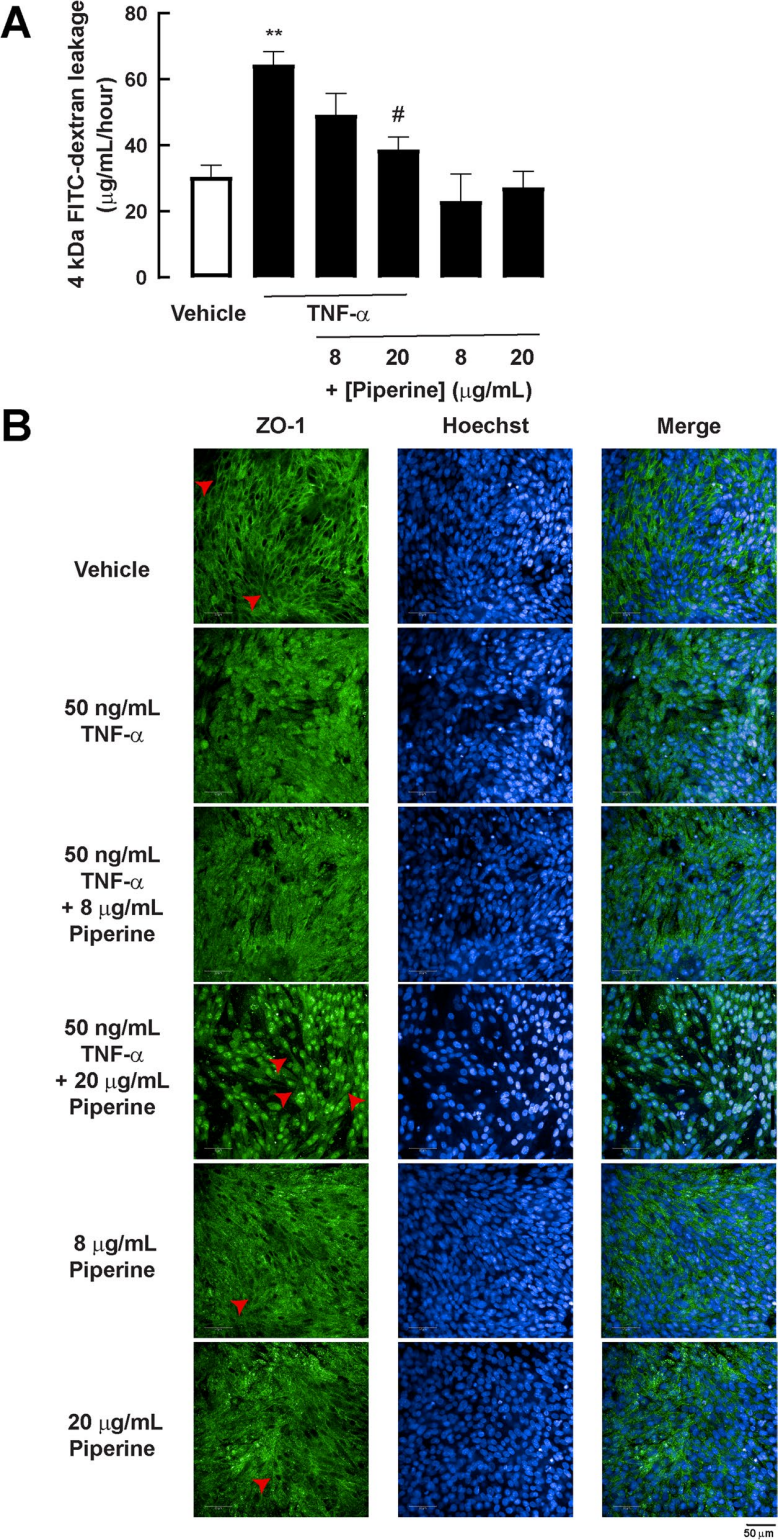
Effect of piperine on TNF-α-induced intestinal barrier defects in 2D porcine duodenal enteroid monolayer. Cells were treated with 50 ng/mL TNF-α with or without 8 or 20 μg/mL piperine for 24 hours. **a** For FITC-dextran experiment, cells were then treated with 15 μL 4 kDa FITC-dextran for 1 hour. Basolateral medium was collected. The concentration of FITC-dextran in the cell culture medium at the basolateral side was calculated according to standard curve of FITC-dextran. (*n* = 3) (**: *p*-value < 0.01 compared with vehicle, #: *p*-value < 0.05 compared with 50 ng/mL TNF-α). **b** For ZO-1 localization, cells were stained with rabbit ZO-1 antibody, following with Alexa Fluor 488 anti-rabbit IgG and Hoechst as nuclear staining. Red arrowheads indicate a linear form of ZO-1 localization

It is known that TNF-α triggers intestinal inflammation, leading to intestinal barrier disruption in weaning piglets [25, 26]. The 2D porcine enteroid monolayer exposed to TNF-α (50 ng/mL) for 24 h with or without co-treatment with piperine was measured for the flux of FITC-dextran (4 kDa), an indicator of intestinal barrier leakage, to determine the effect of piperine on preventing TNF-α-induced intestinal barrier dysfunction. It was found that TNF-α (50 ng/mL; 24 h) induced increased flux of FITC-dextran indicating intestinal barrier leakage (Fig. 5A). The TNF-α-induced barrier leakage was suppressed by co-treatment with piperine in a concentration-dependent manner with a significant effect (~ 70% inhibition) being observed at 20 μg/mL (Fig. 5A). Furthermore, localization of ZO-1, a tight junction protein, was determined using immunofluorescence staining. The linear alignment of ZO-1 indicating normal tight junction integrity was found in the vehicle, whereas the irregular form of ZO-1 localization was found in enteroids exposed to TNF-α (50 ng/mL; 24 h) (Fig. 5B). Interestingly, piperine (20 μg/mL) recovered the TNF-α-induced mislocalization of ZO-1 (Fig. 5B). Since piperine exerted all previous biological activities at 20 μg/mL, next experiments were done using this concentration.

### Effect of piperine on fluid secretion in a 3D model of porcine swelling assay

**Fig. 6:**
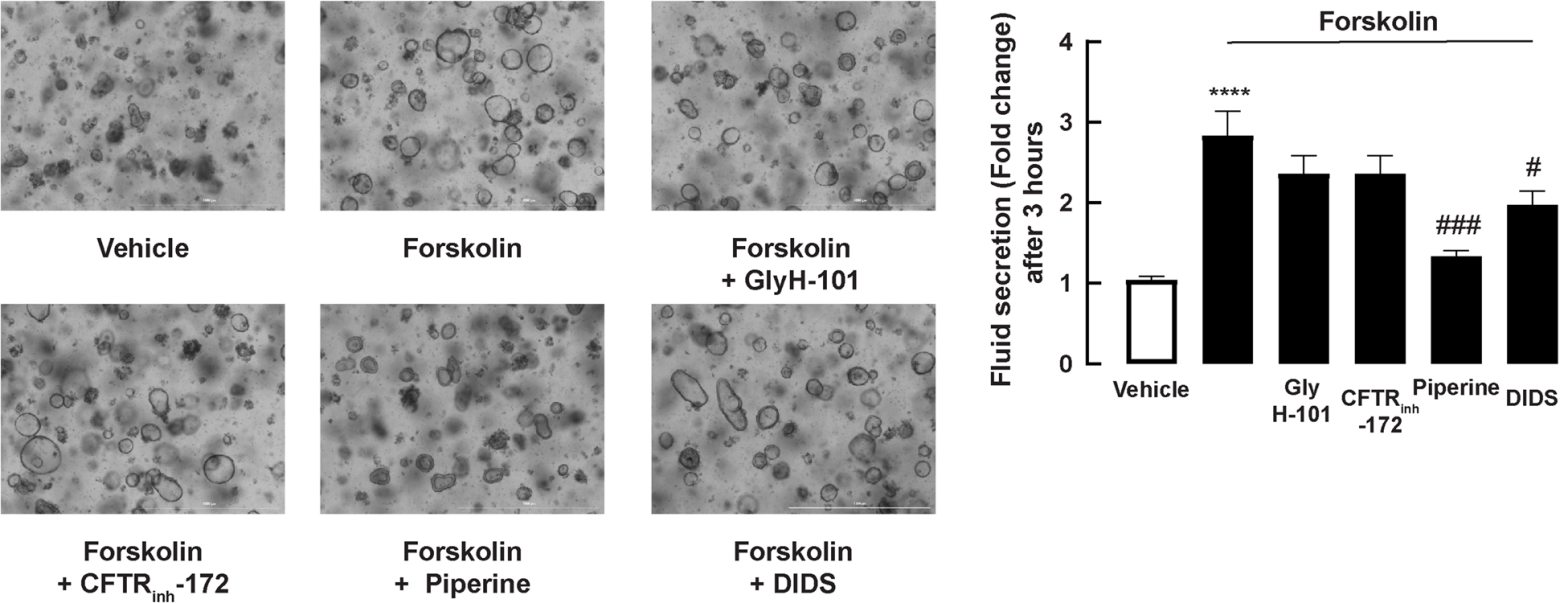
Effect of piperine on CFTR-mediated fluid secretion in forskolin-induced swelling assay. 3D porcine duodenal enteroids were seeded on 48-well plate. Cells were then treated with piperine, CFTR_inh_-172, GlyH-101, or DIDS for 1 hour prior to stimulate with 5 μM forskolin. (Left panel) Representative images of 3D porcine duodenal enteroids after 3 hours treated with 5 μM forskolin (Right panel) Quantitative data of enteroid area after 3 hours treated with 5 μM forskolin. Enteroids areas were measured at least 10 enteroids per 1 sample groups (*n* = 5) (****: *p*-value < 0.0001 compared with vehicle, #: *p*-value < 0.05, ###: *p*-value < 0.001 compared with 5 μM forskolin)

**Fig. 7:**
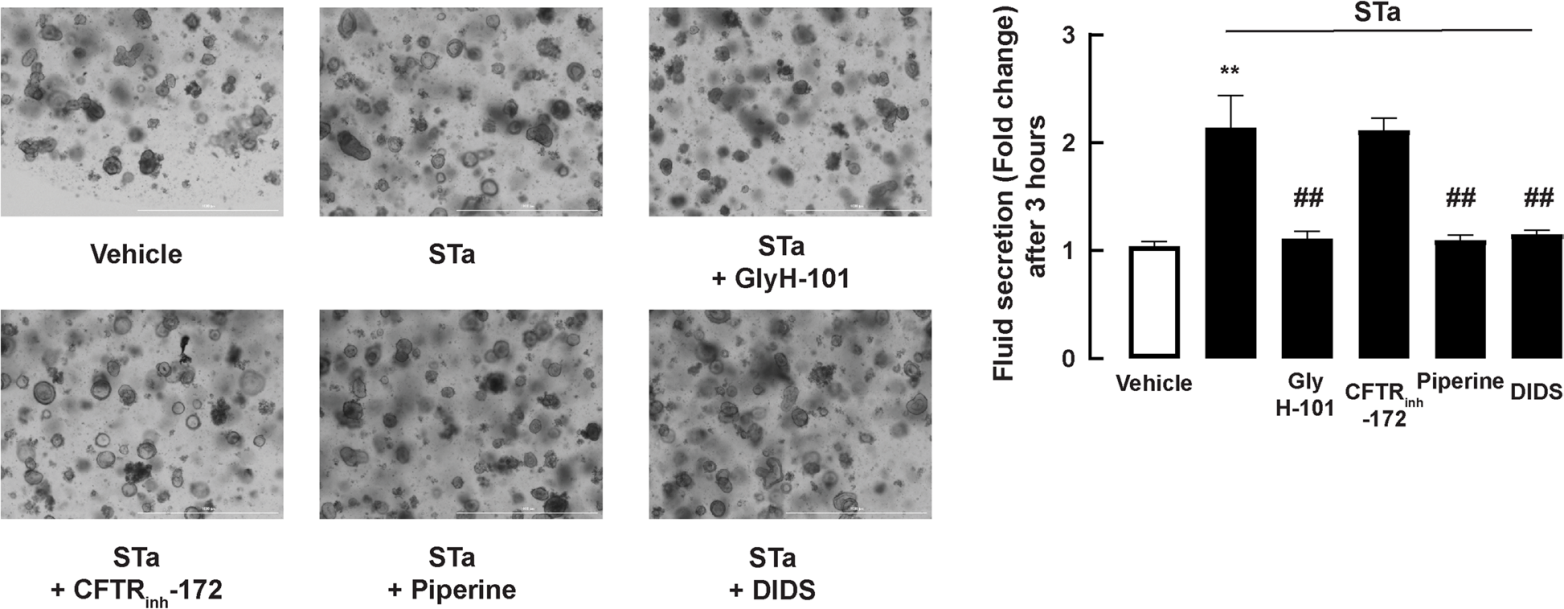
Effect of piperine on CFTR-mediated fluid secretion in STa toxin-induced swelling assay. 3D porcine duodenal enteroids were seeded on 48-well plate. Cells were then treated with piperine, CFTR_inh_-172, GlyH-101, or DIDS for 1 hour prior to stimulate with 100 nM STa toxin. (Left panel) Representative images of 3D porcine duodenal enteroids after 3 hours treated with 100 nM STa toxin (Right panel) Quantitative data of enteroid area after 3 hours treated with 100 nM STa toxin. Enteroids areas were measured at least 10 enteroids per 1 sample groups (*n* = 5) (**: *p*-value < 0.01 compared with vehicle, ##: *p*-value < 0.01 compared with 100 nM STa toxin)

Weaning pigs develop secretory diarrhea as a result of *Escherichia coli* infection via mechanisms involving cAMP/cGMP-dependent chloride and fluid secretion, which is induced by *E. coli*-derived enterotoxins, especially heat-stable enterotoxin (STa) [27]. Furthermore, we used a 3D model of a porcine swelling experiment to examine whether piperine could suppress cAMP and STa-induced fluid assay. The 3D porcine enteroids were pretreated for an hour with piperine or other chloride channel inhibitors before 3-h incubation with forskolin (an adenylate cyclase activator) or STa toxin. As shown in Fig. 6 and Additional file 1: Supplemental video 1; Additional file 2: Supplemental video 2; Additional file 3: Supplemental video 3; Additional file 4: Supplemental video 4; Additional file 5: Supplemental video 5; and Additional file 6: Supplemental video 6, forskolin (5 μM) induced fluid secretion, which was inhibited by piperine (20 μg/mL) and DIDS (200 μM; non-specific chloride channel blocker), and not by known CFTR inhibitors GlyH-101 (50 μM) and CFTR_inh_-172 (20 μM). Interestingly, the STa-induced fluid secretion was inhibited by piperine, GlyH-101, and DIDS, but not by CFTR_inh_-172 (Fig. 7 and Additional file 7: Supplemental video 7; Additional file 8: Supplemental video 8; Additional file 9: Supplemental video 9; Additional file 10: Supplemental video 10; Additional file 11: Supplemental video 11; and Additional file 12: Supplemental video 12).

## Discussion

In this study, we successfully established a porcine duodenal enteroid model derived from intestinal crypts of weaning piglets. This newly established model was used to investigate the effects of piperine on biological activities related to the pathophysiology of post-weaning diarrhea (PWD). In 2D porcine enteroid models, piperine was shown to have anti-oxidative properties against H_2_O_2_ as well as anti-inflammatory effects. Piperine inhibited TNF-α-induced mRNA expression of proinflammatory cytokines without inhibiting NF-κB nuclear translocation and suppressed TNF-α-induced barrier disruption. Interestingly, piperine inhibited forskolin and STa-induced fluid secretion in a 3D model of porcine duodenal enteroid.

We found that piperine inhibited H_2_O_2_-induced oxidative stress in porcine enteroids. It is like that piperine directly scavenges ROS because its co-treatment with H_2_O_2_ produced near complete inhibition of ROS generation, similar to Trolox, which is a direct scavenger of ROS. This notion is in agreement with several previous studies that have demonstrated that piperine acts as a direct scavenger of ROS [28–30]. Likewise, it has been demonstrated that TNF-α-induced ROS was diminished by piperine in weaned Wuzhishan piglets [31]. Results from our investigation provide conclusive evidence that piperine has an anti-oxidative effect on porcine intestinal epithelial cells.

The anti-inflammatory effect of piperine against TNF-α-induced inflammatory responses was evaluated in porcine enteroid models. We found that piperine did not inhibit TNF-α induced NF-κB nuclear translocation. However, piperine was found to inhibit NF-κB nuclear translocation in endothelial cells [32]. The fact that piperine has a cell-type-specific impact on NF-κB nuclear translocation may account for the opposite result. Nonetheless, we found that piperine at 20 μg/mL downregulated TNF-α-induced intestinal inflammation in 2D porcine duodenal enteroids by inhibiting mRNA expression of proinflammatory cytokines and protein expression of IL-1β, suggesting that piperine affects the TNF-α signaling downstream to NF-κB translocation. Additionally, it was demonstrated that piperine downregulated lipopolysaccharide (LPS)-induced TNF-α, IL-6, IL-1β, and prostaglandin E2 production in BV-2 microglia cells [33]. Several studies demonstrated that piperine decreased the level of IL-1β both in vitro and in vivo [34, 35]. In contrast, piperine did not inhibit TNF-α, IL-1β, and IL-6 in the jejunal and ileal mucosa of weaned Wuzhishan piglets [31]. These results suggest that there are some controversial findings regarding the anti-inflammatory effects of piperine in weaning piglets that require further investigation.

Of particular importance, we found that piperine at 20 μg/mL prevented the TNF-α-induced epithelial barrier disruption in the 2D model of porcine enteroids. Likewise, piperine treatment (20 mg/kg and 40 mg/kg) was found to upregulate protein expression of tight junction proteins, including claudin-1, occludin, and ZO-1 in the large intestine of mice [36]. Notably, various spice constituents are known to differentially influence intestinal barrier integrity. For instance, capsaicin decreased transepithelial electrical resistance (TER), whereas piperine increased TER in HCT-8 cells [37]. Furthermore, it was found that piperine repaired intestinal barrier disruption in obese mice by downregulating TNF-α [38]. These findings imply that piperine might aid in the restoration of intestinal barrier function brought on by inflammatory insults.

Anti-secretory effect of piperine was demonstrated in 3D porcine duodenal enteroids. We found that piperine effectively blocked fluid secretion induced by both forskolin and STa. In contrast to piperine and DIDS (non-specific anion channel inhibitors), CFTR_inh_-172 and GlyH-101 did not inhibit fluid secretion in the forskolin-induced swelling assay. In the STa-induced swelling assay, piperine, GlyH-101, and DIDS inhibited fluid secretion, while CFTR_inh_-172 had no effect. The lack of reaction of CFTR_inh_-172 may be because CFTR_inh_-172 had no effect on porcine CFTR, which was reported in preceding studies [39–41]. It is noteworthy that fluid secretion induced by forskolin is more than STa at 3 h of incubation. This may be because there are additional mechanisms contributing to forskolin-induced enteroid swelling, i.e., inhibition of Na^+^/fluid absorption or stimulation of additional chloride secretion pathways, e.g., calcium-dependent chloride secretion [42–44]. This also explains the lack of effect of GlyH-101 CFTR inhibitor on forskolin-induced fluid secretion in this model. Furthermore, GlyH-101 completely inhibited STa-induced fluid secretion, indicating that STa-induced fluid secretion is mainly driven by CFTR-mediated chloride secretion. Piperine inhibited both forskolin and STa-induced fluid secretion, indicating that the efficacy of piperine was not dependent on secretagogues. This is consistent with the previous study reporting that piperine inhibited intestinal chloride secretion in human intestinal epithelial cells (T84 cells) by inhibiting several proteins involved in both cAMP and calcium-dependent chloride secretion including CFTR, calcium-activated chloride channels, and cAMP-dependent K^+^ channels [12].

## Conclusion

In conclusion, piperine exerts anti-oxidative, anti-inflammatory, and anti-secretory effects in porcine duodenal enteroids. The results from this study provide a rational basis for further research and development of piperine or piperine-containing black pepper extract as a functional feed or pharmaceutical agent for the prevention or treatment of PWD.

## Supplementary Information

**Additional file 1: Supplemental video 1**. Time-lapse imaging of enteroid during swelling in vehicle treatment

**Additional file 2: Supplemental video 2**. Time-lapse imaging of enteroid during forskolin-induced swelling in 5 μM forskolin treatment

**Additional file 3: Supplemental video 3**. Time-lapse imaging of enteroid during forskolin-induced swelling in 5 μM forskolin with 50 μM GlyH-101 treatment

**Additional file 4: Supplemental video 4**. Time-lapse imaging of enteroid during forskolin-induced swelling in 5 μM forskolin with 20 μM CFTR_inh_-172 treatment

**Additional file 5: Supplemental video 5**. Time-lapse imaging of enteroid during forskolin-induced swelling in 5 μM forskolin with 20 μg/mL piperine treatment

**Additional file 6: Supplemental video 6**. Time-lapse imaging of enteroid during forskolin-induced swelling in 5 μM forskolin with 200 μM DIDS treatment

**Additional file 7: Supplemental video 7**. Time-lapse imaging of enteroid during swelling in vehicle treatment

**Additional file 8: Supplemental video 8**. Time-lapse imaging of enteroid during STa-induced swelling in 100 nM STa toxin

**Additional file 9: Supplemental video 9**. Time-lapse imaging of enteroid during STa-induced swelling in 100 nM STa toxin with 50 μM GlyH-101 treatment

**Additional file 10: Supplemental video 10**. Time-lapse imaging of enteroid during STa-induced swelling in 100 nM STa toxin with 20 μM CFTR_inh_-172 treatment

**Additional file 11: Supplemental video 11**. Time-lapse imaging of enteroid during STa-induced swelling in 100 nM STa toxin with 20 μg/mL piperine treatment

**Additional file 12: Supplemental video 12**. Time-lapse imaging of enteroid during STa-induced swelling in 100 nM STa toxin with 200 μM DIDS treatment.

**Additional file 13.**

## Data Availability

The data that support the findings of this study are available from the corresponding author [C.M.] upon reasonable request.
