# Supplementary material for: Piperine as potential therapy of post-weaning porcine diarrheas: an in vitro study using a porcine duodenal enteroid model
Source: BMC Vet Res. 2023 Jan 9;19:4. doi: 10.1186/s12917-022-03536-6 (PMC9827699; doi:10.1186/s12917-022-03536-6)
Supplement: Supplementary file 13 — Additional file 13. [file 12917_2022_3536_MOESM13_ESM.pdf]

Supplementary Figure 1

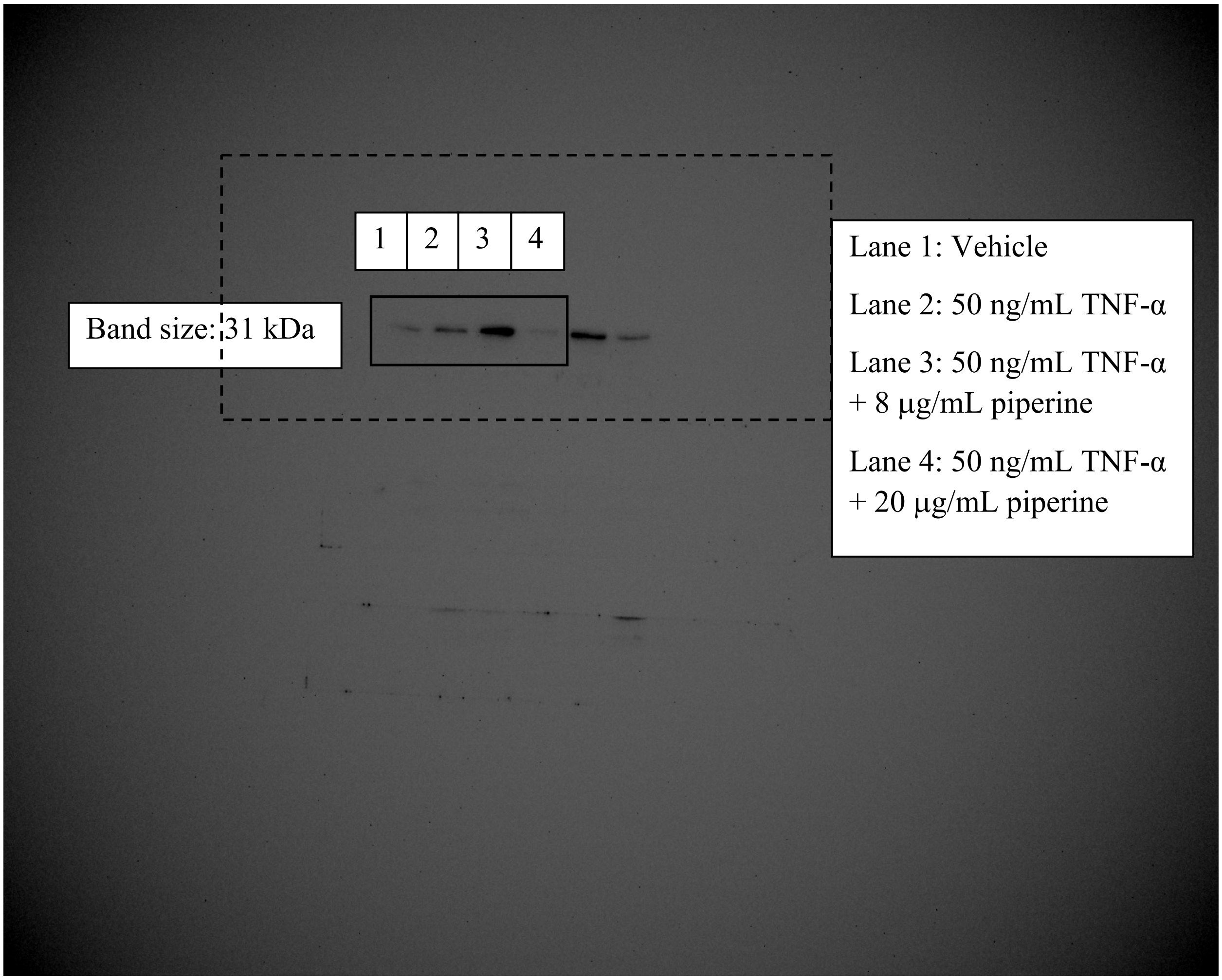

Supplementary Figure 1A. The uncropped image of IL-1β protein expression. The black box determines cropped representative image of IL-1β protein expression as shown in Fig. 4E.

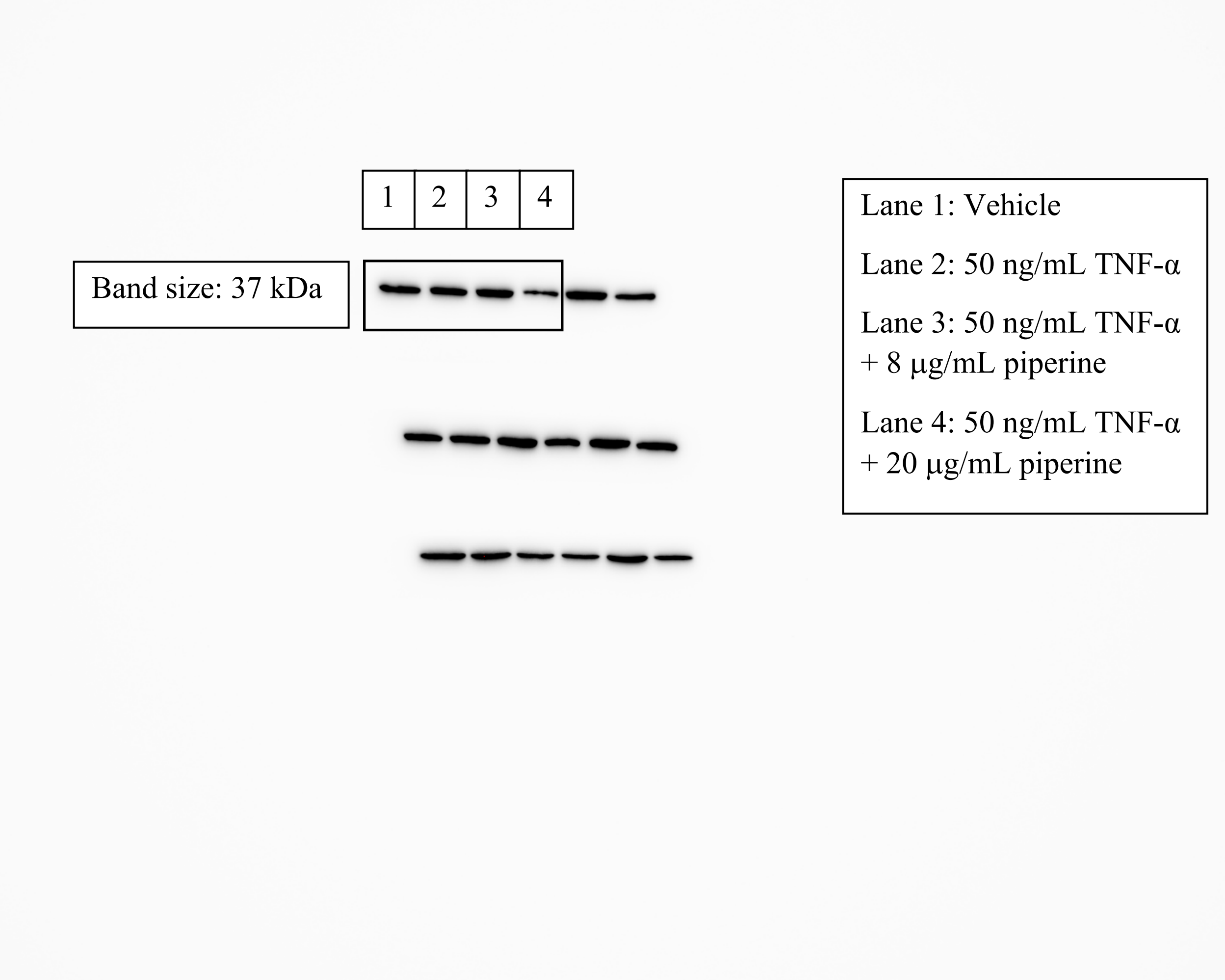
1
2
3
4
Band size: 37 kDa
Lane 1: Vehicle
Lane 2: 50 ng/mL TNF-α
Lane 3: 50 ng/mL TNF-α + 8 μg/mL piperine
Lane 4: 50 ng/mL TNF-α + 20 μg/mL piperine

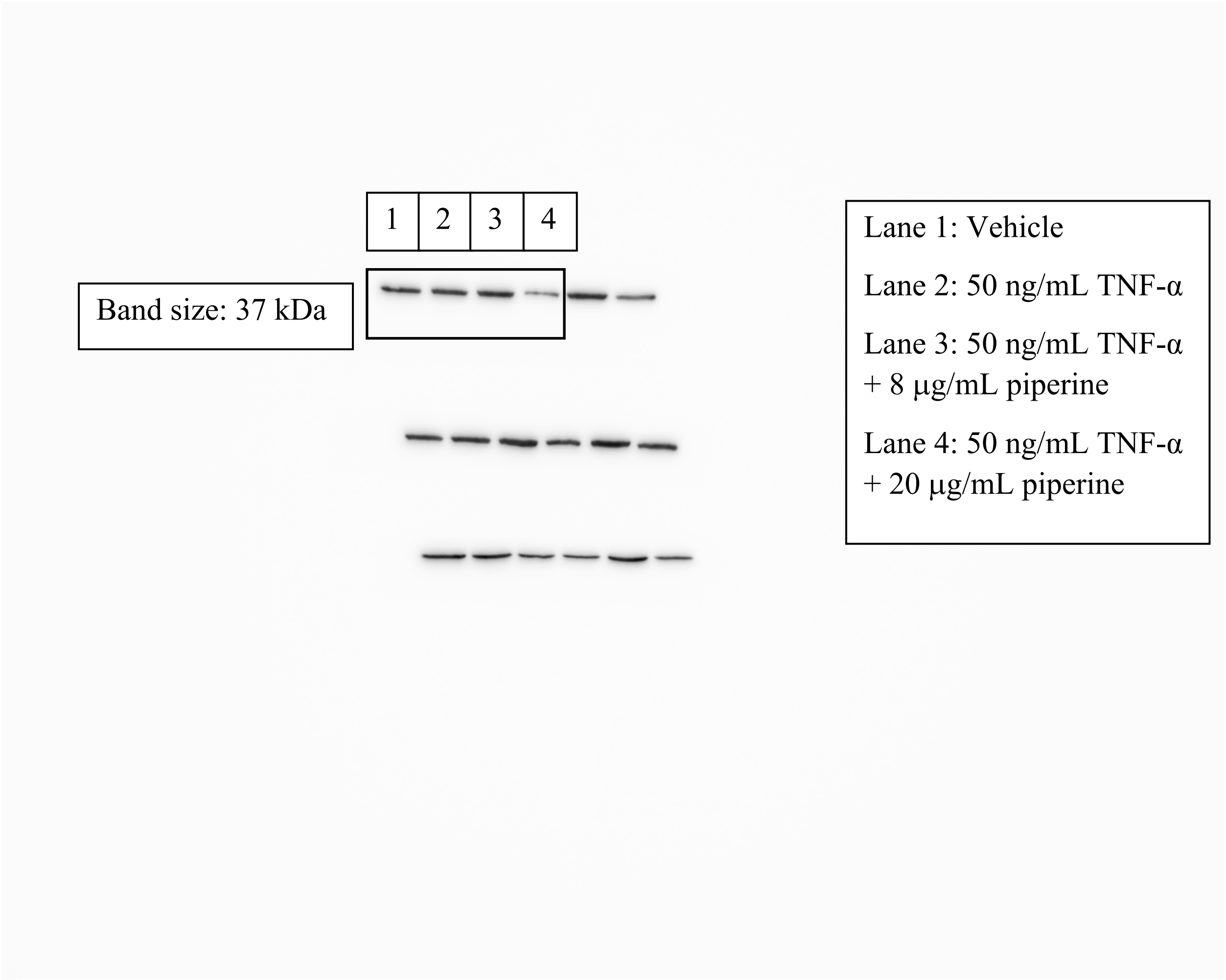

Supplementary Figure 1B. The uncropped raw image of β-actin protein expression showing multiple exposure images from three separate experiments. The black box determines cropped representative image of β-actin protein expression as shown in Fig. 4E.
